# Predictors and Risk Scoring of Postnatal Growth Failure in Very-Low-Birth-Weight Infants

**DOI:** 10.3390/nu18030460

**Published:** 2026-01-30

**Authors:** Nutcha Singhasem, Gunlawadee Maneenil, Anucha Thatrimontrichai, Manapat Praditaukrit, Supaporn Dissaneevate

**Affiliations:** Division of Neonatology, Department of Pediatrics, Faculty of Medicine, Prince of Songkla University, Songkhla 90110, Thailand; nutcha22.naka@gmail.com (N.S.); tanucha@medicine.psu.ac.th (A.T.); manapatpang@gmail.com (M.P.); dsupapor@medicine.psu.ac.th (S.D.)

**Keywords:** extrauterine growth retardation, predictions, postnatal growth failure, preterm infant, risk factors

## Abstract

**Objectives**: To identify factors associated with postnatal growth failure (PGF) in very-low-birth-weight (VLBW) infants and to develop a model for the early identification of neonates at risk. **Methods**: This retrospective cohort study included VLBW infants born between 2014 and 2024. PGF was defined using the 2013 Fenton growth chart. Multivariate logistic regression was used to identify predictors of PGF, and a weighted risk score was derived from their relative contributions. Model performance was evaluated using a receiver operating characteristic (ROC) curve. **Results**: Among 481 VLBW infants, 334 (69.4%) had PGF. Independent predictors were birth weight < 750 g (adjusted odds ratio [aOR] 8.11; 95% confidence interval [CI], 3.01–21.83), birth weight 750–1000 g (aOR 2.39; 95% CI, 1.35–4.21), multiple births (aOR 2.82; 95% CI, 1.71–4.67), pregnancy-induced hypertension (PIH) (aOR 3.32; 95% CI, 2.02–5.46), oligohydramnios (aOR 4.08; 95% CI, 1.68–9.92), no antenatal corticosteroid exposure (aOR 2.97; 95% CI, 1.65–5.36), and formula or mixed feeding (aOR 1.69; 95% CI, 1.08–2.64). The model showed good discrimination for scores ≥2 (area under the ROC curve, 0.736; sensitivity, 71.6%; specificity, 64.5%). **Conclusions**: Birth weight < 1000 g, multiple births, PIH, oligohydramnios, no antenatal corticosteroid exposure, and formula or mixed feeding were significant predictors of PGF. The score may support early risk stratification and prompt closer nutritional surveillance.

## 1. Introduction

Recent advancements in neonatal care, including improved respiratory support, parenteral nutrition, and infection control, have significantly increased the survival rate of preterm infants. Despite these improvements, morbidity and adverse neurodevelopmental outcomes remain significant challenges. Postnatal growth plays a critical role in long-term neurodevelopment because early growth restriction is associated with poor cognitive outcomes and increased metabolic risk in later life [1,2,3,4].

Postnatal growth failure (PGF) remains a prevalent concern among preterm infants, particularly those with extremely low birth weight [4]. PGF is closely related to extrauterine growth restriction (EUGR), although the terms are not fully synonymous. EUGR typically refers to a statistical definition based on anthropometric percentiles, whereas PGF is used clinically to reflect inadequate postnatal growth. PGF is a significant condition associated with short-term morbidities, including nosocomial infections, patent ductus arteriosus (PDA), and bronchopulmonary dysplasia (BPD) [5], as well as long-term consequences such as impaired growth and neurodevelopmental delays [1,6]. In addition, prior studies have reported associations between PGF and an increased risk of cardiovascular disease and metabolic syndrome in childhood [7,8].

Despite the recognized impact of PGF, there is no universally accepted diagnostic criterion for its identification [9]. In the present study, we adopted a definition of weight <10th percentile at discharge or 36–40 weeks postmenstrual age using the Fenton growth chart, consistent with the statistical criteria commonly used to describe EUGR in contemporary studies [10,11,12,13]. This statistical definition should be distinguished from the clinical concept of PGF, which reflects a growth trajectory influenced by nutritional adequacy, comorbidities, and metabolic needs. Accordingly, throughout this study, PGF refers specifically to the statistical EUGR endpoint. Clarifying this distinction is essential for interpreting the target of the predictive model and for enabling comparison with the prior literature.

Although current guidelines emphasize adequate energy intake and nutritional strategies to promote growth similar to that of a fetus and monitoring weight gain to parallel fetal growth at the same gestational age [14,15], the incidence of PGF remains high and varies significantly across studies [13,16,17]. Early identification of infants at high risk of PGF is clinically desirable, as it may enable clinicians to implement nutritional and medical interventions during a critical period of neurodevelopment. Although numerous perinatal and clinical risk factors for impaired postnatal growth are well characterized, they are rarely integrated into practical screening tools for early risk stratification in routine care. Accordingly, this study aimed to identify antenatal and early postnatal factors associated with PGF as defined by statistical criteria and to develop a pragmatic risk-scoring model to support early nutritional risk stratification in VLBW infants. Rather than proposing new mechanistic predictors, the model integrates readily available clinical variables used in routine neonatal care and may be particularly applicable in resource-limited Neonatal Intensive Care Unit (NICU) settings.

## 2. Materials and Methods

### 2.1. Study Design

This retrospective cohort study was conducted between January 2014 and December 2024 at the Level IV NICU of Songklanagarind Hospital, a tertiary referral and university hospital in southern Thailand with approximately 2300 annual births. Neonates with a birth weight < 1500 g admitted to the NICU were eligible. Exclusion criteria included outborn infants, those with chromosomal abnormalities or major congenital anomalies, and infants who died or were transferred before discharge or before reaching 36 weeks postmenstrual age.

This study was approved by the Institutional Review Board and Ethics Committee of the Faculty of Medicine, Prince of Songkla University, Thailand (Approval No. REC 67-046-1-1). The requirement for obtaining informed consent was waived because the study was retrospective in nature.

### 2.2. Nutrition Management in Very-Low-Birth-Weight Infants

Parenteral nutrition (PN) was initiated using total parenteral nutrition (TPN) or a starter solution within the first 24 h of life. The starter solution (100 mL) contained 3 g/kg/day of amino acids, 12.5% dextrose, and 1.5 mmol of calcium. TPN was introduced as soon as possible, and dextrose was infused at a rate of 4–8 mg/kg/min. Amino acids were started at 1.5–2 g/kg/day and increased by 0.5–1 g/kg/day to a maximum of 3.5–4 g/kg/day. Lipids were supplemented with soybean oil, medium-chain triglycerides, olive oil, and fish oil (SMOF) lipid (Fresenius Kabi Austria GmbH, Graz, Austria) containing fish oil (3 g/100 mL), medium-chain triglycerides (6 g/100 mL), olive oil (5 g/100 mL), and soybean oil (6 g/100 mL). The initial SMOF lipid dose was 1.5–2 g/kg/day, increasing by 0.5–1 g/kg/day to a maximum of 4.0 g/kg/day. PN was discontinued when the enteral feeding volume exceeded 120 mL/kg/day.

Once the infant’s hemodynamic condition stabilized, enteral nutrition (EN) was initiated, typically within 24–48 h of life. Minimal enteral feeding began at 10–20 mL/kg/day and was increased by 15–30 mL/kg/day, depending on the infant’s body weight. The feeding volume was gradually increased to 150–180 mL/kg/day or until energy intake reached 115–140 kcal/kg/day [14]. Expressed breast milk (EBM) or donor milk (DM) is the preferred choice for initiating feeding. When enteral feeding with EBM or DM reached 80–120 mL/kg/day, fortification was introduced using preterm formula powder or concentrated preterm formula to achieve 25 kcal/oz and 30 kcal/oz, respectively. If EBM was unavailable and parents declined consent for DM, a premature formula was used.

### 2.3. Data Collection

Maternal and neonatal information were retrieved from electronic medical records in the Hospital Information System (HIS) database. Maternal data included age, underlying conditions, antenatal care complications, and corticosteroid exposure. Neonatal demographic data included gestational age, sex, birth weight, 1 and 5 min Apgar scores, and multiple birth statuses. Additionally, we reviewed PN, EN, and growth parameters, such as body weight, head circumference, and length, recorded in the HIS database.

Milk feeding during hospitalization was categorized into three groups based on the predominant type (>50% of the total milk volume): EBM/DM with fortification, infant formula, or a mixture. We reviewed the age at initiation and achievement of full enteral feeding and observed that enteral intake exceeded 100 mL/kg/day or 120 kcal/kg/day, PN duration, and day of regaining birth weight.

Neonatal illnesses and morbidities recorded for each infant included respiratory distress syndrome (RDS), hemodynamically significant PDA requiring medication or surgical treatment, and necrotizing enterocolitis defined by the modified Bell’s criteria [18]. Sepsis was categorized as clinical or culture-proven, and anemia requiring blood transfusion was documented. Other conditions included BPD based on the National Institute of Child Health and Human Development criteria [19], retinopathy of prematurity, intraventricular hemorrhage (IVH), and periventricular leukomalacia. Additionally, we reviewed the duration of oxygen supplementation, invasive ventilation, length of hospital stay, and overall hospitalization cost.

### 2.4. Definitions

Small for gestational age (SGA) was defined as a birth weight below the 10th percentile according to the Fenton & Kim (2013) growth chart [10]. PGF was diagnosed when the weight was below the 10th percentile at either approximately 36–40 weeks of postmenstrual age or at discharge [11,20]. In this study, we used the Fenton growth chart as a reference to diagnose PGF [10]. Pregnancy-induced hypertension was diagnosed according to the American College of Obstetricians and Gynecologists criteria [21]. Oligohydramnios was defined as a single deepest vertical pocket of <2 cm on ultrasonography, amniotic fluid volume of <5% for gestational age, an amniotic gestational age, or an amniotic fluid index of <5 cm [22]. Exposure to antenatal corticosteroids was defined as maternal receipt of at least one dose of corticosteroids [23].

### 2.5. Statistical Analysis

Categorical variables were expressed as percentages and compared using the Chi-square or Fisher’s exact test. Continuous variables were reported as mean ± standard deviation (SD) or median (interquartile range; IQR) and compared using Student’s *t*-test or Wilcoxon’s rank-sum test. Univariate and multivariate logistic regression analyses identified factors associated with PGF, with variables meeting *p* < 0.2 in univariate analysis included in a backward stepwise multivariate model. aORs and 95% confidence intervals (CIs) were reported for significant predictors.

A PGF predictive model was developed by transforming β coefficients from logistic regression into risk scores. Multicollinearity was assessed using variance inflation factors for each parameter. Model discrimination was evaluated using receiver operating characteristic (ROC) curve analysis, with the area under the ROC curve (AUC) used to determine the optimal cut-off. Sensitivity and specificity were calculated to assess predictive accuracy, and model calibration was evaluated using the Hosmer–Lemeshow goodness-of-fit test. Internal validation was performed using bootstrapping with 500 resamples [24]. Statistical significance was defined as *p* < 0.05. All analyses were conducted using the *Epicalc* package in R (version 4.4.1; R Foundation for Statistical Computing, Vienna, Austria).

## 3. Results

One thousand and twenty-four VLBW infants were admitted to our NICU during the study period, of whom 481 met the inclusion criteria and were included in the study, while 543 infants were excluded for the following reasons: birth outside the hospital (*n* = 301), death or referral before discharge, postmenstrual age of 36 weeks (*n* = 202), or chromosomal or major congenital abnormalities (*n* = 40). The median (IQR) gestational age and birth weight were 30 (28, 31) weeks and 1166 (885, 1375) g, respectively. The distribution of gestational age categories was as follows: 111 infants (23.1%) were born at 23–27 weeks, 252 (52.4%) at 28–31 weeks, and 118 (24.5%) at ≥32 weeks.

PGF was diagnosed in 334 infants (69.4%), including all infants classified as SGA (*n* = 151). The clinical characteristics of the PGF and non-PGF groups are presented in Table 1. Birth and discharge weights were significantly lower in the PGF group than those in the non-PGF group. No statistically significant differences were noted in head circumference or length at birth between the two groups. Moreover, air leak syndrome, pulmonary hemorrhage, hypotension requiring >2 agents, and anemia requiring blood transfusion were not significantly different between the two groups.

All infants received PN within the first 24 h of age (median [IQR] 3 [1, 9] h), and the median (IQR) duration of PN was 13 (8, 25) days. The overall median (IQR) ages at the first start and full enteral feeding were 2 (2, 4) and 14 (9, 22) days, respectively. Approximately 40% and 15% of infants received EBM and DM, respectively, as their initial enteral feed. Fifty-eight percent of all infants received EBM or DM as an enteral feed during hospitalization. Statistically significant differences were noted between the PGF and non-PGF groups in the duration of PN, day of reaching a body weight greater than the birth weight, and type of milk feeding during hospitalization. Other nutritional management strategies were compared between the two groups and are presented in Table 2.

Other outcomes, including the duration of oxygen use and invasive ventilation, length of hospital stay, hospital costs, and mortality, were not significantly different between the two groups. The overall mortality rate in this study was 2.1% (10/481).

The factors associated with PGF are presented in Table 3. Multivariate logistic regression analysis identified birth weight < 1000 g, multiple births, PIH, oligohydramnios, lack of antenatal corticosteroid exposure, and infant formula or mixed feeding as significant risk factors for PGF.

**Figure 1:**
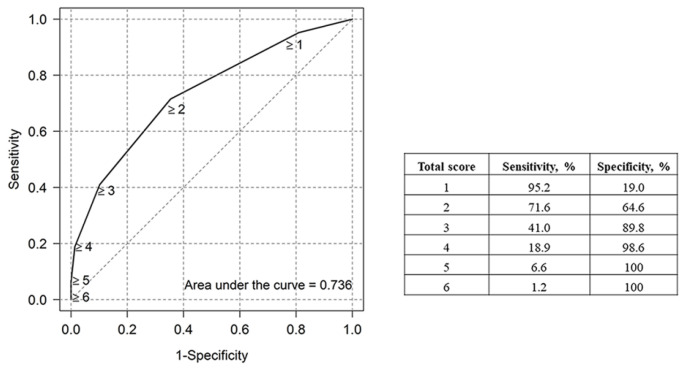
ROC curve of clinical risk score on the prediction of postnatal growth failure in very-low-birth-weight infants.

A clinical prediction tool was developed based on the β coefficients of the six identified risk factors (Table 4). A birth weight of <750 g and oligohydramnios had the highest β coefficient values and were assigned a score of 2, while the remaining factors were assigned a score of 1. A risk score of ≥2 was designated the optimal cut-off for predicting PGF, with a sensitivity of 71.6% and specificity of 64.6%, and the AUC of the model was 0.736 (95% CI: 0.732−0.740) (Figure 1). The goodness-of-fit of the model was confirmed by the Hosmer–Lemeshow test, with a *p*-value of 0.551. Internal validation using the bootstrapping method (500 times) was performed, resulting in an observed-to-expected ratio of 1.000 and a C-statistic of 0.757 (95% CI: 0.753–0.761). All values were within the expected range [24].

## 4. Discussion

PGF is common among VLBW infants and remains a major challenge despite advances in neonatal care and nutrition. In this single-center cohort, PGF was diagnosed using the Fenton & Kim [10] growth chart and was observed in 69.4% of VLBW infants. The score-based model developed in this study incorporated six factors and demonstrated moderate predictive performance (AUC 0.736).

The incidence of PGF varies depending on the diagnostic criteria and study settings. Using the criterion of discharge weight of <10th percentile on the Fenton chart, reported incidences ranged from 47 to 87% in China [25,26] and 24% to 60% in Europe and the USA [13,17,27].

Our predictive model incorporated six factors: PIH, lack of antenatal corticosteroid exposure, multiple births, oligohydramnios, birth weight < 1000 g, and infant formula or mixed feeding. The model uses variables accessible during routine care and may assist clinicians in early risk stratification.

We defined PGF using the 2013 Fenton growth chart, which is practical and widely used in the NICU. However, in preterm infants, the Fenton chart represents an intrauterine growth curve rather than an extrauterine postnatal growth curve, and may introduce conceptual limitations that could affect model validity. Reliance on a single weight-based criterion may not fully capture longitudinal growth dynamics, such as growth velocity or changes in z scores. In addition, a substantial proportion of infants classified as PGF were already SGA at birth, highlighting conceptual overlap between antenatal and postnatal growth restriction. This overlap may partially inflate the predictive contribution of birth weight–related variables and should be considered when interpreting model performance. Future studies incorporating longitudinal growth velocity or body composition measures may provide a more comprehensive assessment.

Gao et al. [26] developed a predictive model for PGF in preterm infants (<32 weeks of gestation) using a Fenton growth chart. Their model included eight predictors: birth weight, SGA, hypertensive disease complicating pregnancy, gestational diabetes mellitus (GDM), multiple births, cumulative fasting duration, growth velocity, and postnatal corticosteroid use. They constructed a nomogram for PGF risk probabilities with an AUC of 83%. Additionally, two previous studies from Korea developed machine learning models to predict PGF at different time points, including 0, 7, 14, and 28 days after birth, and at discharge. These models demonstrated good predictive performance for PGF in VLBW infants [28,29]. Previous studies have reported other factors associated with PGF. Zhao et al. reported that low gestational age, intrauterine growth restriction, RDS, and necrotizing enterocolitis (NEC) are risk factors for PGF [25]. Moreover, the number of days to regain birth weight, age at initiation, achievement of full enteral feeding, and duration of PN contribute to the risk of PGF [5,12,30].

In this study, several nutrition-related and time-dependent variables showed significant associations with PGF in univariate analyses; however, these associations attenuated after adjustment for upstream perinatal predictors in multivariable models. This pattern suggests mediation rather than independent prediction. Consistent with this interpretation, VIF values for these variables were <10, indicating no substantial multicollinearity. Accordingly, their exclusion was based on considerations of parsimony and causal structure rather than collinearity.

The association between formula or mixed feeding and PGF likely reflects illness severity rather than a direct causal effect. Infants with feeding intolerance or significant morbidities are more likely to receive non-exclusive human milk, resulting in confounding by indication. Prior studies have reported inconsistent associations between feeding type and PGF [12,16], partly owing to variability in human milk fortification practices and DM availability. During the COVID-19 pandemic, reduced maternal presence and infection control restrictions may have further limited breastfeeding and influenced observed feeding patterns. Moreover, the relationship among human milk intake, fortification strategies, and postnatal growth remains controversial. Although exclusive human milk feeding has been associated with improved long-term outcomes, growth faltering in the absence of adequate fortification is common. Consistent with the existing literature, these findings reflect this ongoing tension and should not be interpreted as evidence against human milk feeding; rather, they underscore the importance of individualized nutritional support.

One of the PGF predictors in our study was PIH, which is consistent with the findings of a previous study [26]. Pregnant women with PIH experience reduced placental perfusion due to increased resistance in small arteries and a lack of pro-angiogenic factors, which limits nutrient exchange, leading to poor intrauterine growth and potentially exacerbating preterm birth [31,32].

This study observed that multiple births were significantly associated with PGF, which is consistent with the findings of previous studies [2,26]. Infants from mothers with multiple pregnancies have a high risk of obstetric complications, including fetal anomalies, growth abnormalities, and selective intrauterine growth restriction, which can lead to preterm birth and increased morbidity and mortality [33]. Lee et al. [23] reported that the rate of oligohydramnios was significantly higher in infants with PGF than that in non-PGF infants, which is consistent with our findings. Oligohydramnios is associated with pregnancy complications, such as GDM, uteroplacental insufficiency, chronic hypoxia, and premature rupture of membranes [34]. A systematic review and meta-analysis by Rabie et al. [22] reported that pregnant females with comorbidities and oligohydramnios are more likely to have low-birth-weight infants, which is a potential risk factor for PGF.

Our study observed that a lack of antenatal corticosteroid administration was a risk factor for PGF, consistent with the findings of Clark et al. [11]. Previous studies have revealed that corticosteroids promote lung maturation through multiple mechanisms, including stimulation of surfactant production, increased phospholipid synthesis, activation of antioxidant enzymes, and inhibition of DNA synthesis [35]. Furthermore, previous studies have reported low rates of morbidities, including RDS, IVH, and NEC, and improved survival in infants exposed to antenatal corticosteroids, which may contribute to reducing the incidence of PGF [36].

While the predictors included in this model have been previously described, our aim was to translate them into a simplified bedside screening tool for early identification of PGF risk rather than to provide new mechanistic insights. Although a cut-off of ≥2 points provided optimal discrimination in our cohort, classification errors persisted. False negatives represent infants who ultimately develop PGF despite scores below the threshold, whereas false positives represent infants who do not develop PGF but meet the cut-off based on antenatal or perinatal characteristics. In clinical practice, false positives may prompt increased nutritional monitoring or earlier consideration of fortification strategies, which are generally low risk; thus, their clinical consequences are likely modest. False negatives may be more concerning because failure to identify at-risk infants can delay nutritional intensification during a critical window for postnatal growth and neurodevelopment. These considerations underscore that the proposed score should be viewed as a screening tool to support early risk stratification rather than a standalone diagnostic instrument. Longitudinal growth monitoring and nutritional assessment remain necessary for individualized decision-making. In addition, the optimal threshold may vary with baseline PGF prevalence and unit-specific nutritional practices, suggesting that local calibration or external validation is needed before implementation in other NICU settings.

Our study had several limitations. First, PGF was defined using a single statistical cut-off at 36–40 weeks PMA or discharge, which may misclassify infants with divergent growth trajectories and may not fully capture the clinical construct of PGF. Second, infants who died or were transferred before outcome ascertainment were excluded, introducing survivorship bias and limiting applicability to VLBW survivors; consequently, the effects of morbidities such as BPD, NEC, and sepsis may have been underestimated. Third, although neonatal nutritional protocols were described, detailed data on protein–energy intake, feeding interruptions, and metabolic tolerance were unavailable, limiting biological interpretability. Fourth, the model was developed using backward stepwise selection, which carries a risk of overfitting in the absence of predefined predictors. Although internal validation was performed, external validation was not, and generalizability to NICUs with different feeding practices remains uncertain. Fifth, the retrospective single-center design spanning 10 years may introduce temporal bias due to evolving NICU and feeding practices, including changes during the COVID-19 pandemic that affected breastfeeding rates and DM access. Taken together, the proposed score should be viewed as a pragmatic early screening tool rather than a definitive prognostic instrument and requires multicenter validation before clinical integration.

Despite these limitations, the prediction score is simple, does not require laboratory or imaging data, and uses information routinely available at or shortly after birth. Early identification of infants at risk of PGF may support targeted nutritional interventions and individualized monitoring. Future research should evaluate the clinical consequences of misclassification and examine associations with long-term neurodevelopmental and metabolic outcomes.

## 5. Conclusions

PGF remains a significant concern in VLBW infants, particularly among those with antenatal and perinatal risk factors. Using a statistical definition at a single time point, our model showed moderate performance in identifying infants at increased risk. While the score may support early risk stratification and prompt closer nutritional surveillance, it should be used as a pragmatic screening tool rather than a therapeutic decision instrument. External validation and incorporation of longitudinal growth trajectories are essential to refine predictive performance and determine clinical applicability.

## Figures and Tables

**Table 1:** Clinical characteristics between the postnatal growth failure and non-postnatal growth failure groups.

Characteristics	PGF Group (*n* = 334)	Non-PGF Group (*n* = 147)	*p*-Value
Gestation age, weeks	30.5 (28, 32)	29 (27, 30)	<0.001
Birth weight, g	1100 (815, 1360)	1275 (1032, 1392)	<0.001
≤750	62 (18.6)	5 (3.4)	<0.001
751–1000	77 (23.1)	28 (19)	
1001–1250	81 (24.3)	39 (26.5)	
1251–1500	114 (34.1)	75 (51)	
Cesarean section	273 (81.7)	97 (66)	<0.001
Antenatal corticosteroid exposure	252 (75.4)	128 (87.1)	0.006
PIH	140 (41.9)	33 (22.4)	<0.001
Multiple gestation	111 (33.2)	35 (23.8)	0.04
Oligohydramnios	47 (14.1)	7 (4.8)	0.006
RDS	248 (74.3)	132 (89.8)	<0.001
RDS requiring surfactant	120 (48.4)	77 (58.3)	0.08
hsPDA	128 (38.3)	65 (44.2)	0.27
required medical treatment	101 (78.9)	53 (81.5)	0.81
required surgical treatment	14 (10.9)	1 (1.5)	0.04
NEC at all stages	69 (20.7)	20 (13.6)	0.09
Stages 2 and 3	38 (55.1)	10 (50)	0.88
Proven sepsis	53 (15.9)	18 (12.2)	0.37
BPD	159 (47.6)	78 (53.1)	0.32
IVH grade III and IV	14 (4.2)	10 (6.8)	0.37
PVL	17 (5.1)	8 (5.4)	0.99
ROP	72 (21.6)	18 (12.2)	0.02
Duration of invasive ventilation, day	2 (0, 15.2)	3 (1, 9)	
Weight at discharge, g	1880 (1690, 2280)	2370 (2085, 2746)	<0.001
Length of stay, day	50 (26, 75)	51 (37, 69)	0.28
Hospital cost, US$	7743 (3412, 13,243)	7701 (4927, 11,705)	0.88

Data are expressed as n (%), median (IQR). BPD: bronchopulmonary dysplasia; hsPDA: hemodynamically significant patent ductus arteriosus; IVH: intraventricular hemorrhage; NEC: necrotizing enterocolitis; PIH: pregnancy-induced hypertension; PVL: periventricular leukomalacia; RDS: respiratory distress syndrome; ROP: retinopathy of prematurity; IQR: interquartile range; PGF: postnatal growth failure.

**Table 2:** Comparison of nutritional management between the postnatal growth failure and non-postnatal growth failure groups.

Variables	PGF Group (*n* = 334)	Non-PGF Group (*n* = 147)	*p*-Value
Age of start of enteral feeding, day	2 (2, 5)	2 (2, 4)	0.44
Age of full enteral feeding, day	15 (9, 24)	14 (9, 20)	0.13
Initiation of feeding with EBM or DM	170 (50.1)	83 (56.4)	0.16
Age of initiation of parenteral nutrition, h	3 (1, 8.5)	3 (1, 9)	0.63
Initial protein intake, g/kg/day	2 (2, 2)	2 (2, 2)	0.53
Initial fat intake, g/kg/day	2 (1, 2)	2 (1, 2)	0.73
Initial glucose intake, g/kg/day	7.6 (7.1, 8.1)	7.7 (7.2, 8.2)	0.70
Duration of parenteral nutrition, days	14 (8, 28)	12 (7, 19)	0.02
Day of reaching > 100 mL/kg of enteral feeding, day	13 (8, 20)	12 (8, 17.8)	0.22
Day of reaching > 120 kcal/kg of enteral feeding, day	16 (11, 26)	15 (11, 21)	0.10
Day of reaching full enteral feeding, day	15 (9, 24)	14 (9, 20)	0.13
Day of reaching body weight > birthweight, day	10 (7, 13)	8 (6, 12)	0.02
Type of predominant milk feeding, n (%)			0.02
EBM/DM with fortification	179 (53.6)	98 (66.7)	
Infant formula	46 (13.8)	12 (8.2)	
Mixture	109 (32.6)	37 (25.2)	

Data are expressed as *n* (%), median (IQR). EBM: expressed breast milk; DM: donor milk; IQR: interquartile range; PGF: postnatal growth failure.

**Table 3:** Factors associated with postnatal growth failure based on univariate and multivariate analyses.

Variables	Univariate		Multivariate	
	OR (95% CI)	*p*-Value	aOR (95% CI)	*p*-Value
Birthweight, g ref. = 1251–1500				
≤750	8.16 (3.13–21.23)	<0.001	8.11 (3.01–21.83)	<0.001
750–1000	1.81 (1.07–3.05)	0.03	2.39 (1.35–4.21)	0.003
1001–1250	1.37 (0.85–2.21)	0.20	1.5 (0.89–2.51)	0.127
Multiple births	1.59 (1.02–2.48)	0.04	2.82 (1.71–4.67)	<0.001
PIH	2.49 (1.6–3.89)	<0.001	3.32 (2.02–5.46)	<0.001
Lack of antenatal steroid exposure	2.19 (1.27–3.77)	<0.001	2.97 (1.65–5.36)	<0.001
Oligohydramnios	3.35 (1.47–7.59)	0.004	4.08 (1.68–9.92)	<0.001
PDA with surgical treatment	7.86 (1.01–61.16)	0.02	-	-
NEC	1.65 (0.96–2.84)	0.06	-	-
Infant formula and mixture feeding	1.73 (1.16–2.60)	0.007	1.69 (1.08–2.64)	0.02
Age of full enteral feeding	1.03 (1.01–1.04)	0.005	-	-
Duration of parenteral nutrition	1.03 (1.01–1.04)	<0.001	-	-
Day of reaching body weight > birthweight	0.97 (0.93–1.01)	0.09	-	-
Duration of invasive ventilation	1.03 (1.01–1.04)	<0.001	-	-

aOR: adjusted odds ratio; PDA: patent ductus arteriosus; PIH: pregnancy-induced hypertension; NEC: necrotizing enterocolitis; CI: confidence interval.

**Table 4:** Risk scores for the predictive model of postnatal growth failure in very-low-birth-weight infants.

Variables	β Coefficient	Risk Score
Birthweight, g		
≤750	2.093	2
750–1000	0.870	1
1001–1250	0.403	0
Multiple births	1.037	
Yes		1
No		0
Pregnancy-induced hypertension	1.199	
Yes		1
No		0
Type of predominant milk feeding	0.525	
Infant formula/Mixture		1
EBM/DM with fortification		0
Antenatal steroid exposure	1.089	
Yes		0
No		1
Oligohydramnios	1.406	
Yes		2
No		0

## Data Availability

The data presented in this study are available upon reasonable request from the corresponding author. The data are not publicly available due to privacy and ethical restrictions related to the protection of participants’ information.

## Abbreviations

The following abbreviations are used in this manuscript:

DM	Donor milk
EBM	Expressed breast milk
NEC	Necrotizing enterocolitis
PDA	Patent ductus arteriosus
PGF	Postnatal growth failure
PIH	Pregnancy-induced hypertension
RDS	Respiratory distress syndrome
SGA	Small for gestational age
VLBW	Very low birth weight
